# Spatially resolved Raman spectroscopy of defects, strains, and strain fluctuations in domain structures of monolayer graphene

**DOI:** 10.1038/s41598-017-16969-z

**Published:** 2017-11-30

**Authors:** Taegeon Lee, Felisita A Mas’ud, Myung Jong Kim, Heesuk Rho

**Affiliations:** 1 Department of Physics, Research Institute of Physics and Chemistry, Chonbuk National University, Jeonju, 54896 Korea; 2 Applied Quantum Composites Research Center, Korea Institute of Science and Technology, Wanju, 55324 Korea

## Abstract

We report spatially resolved Raman scattering results of polycrystalline monolayer graphene films to study the effects of defects, strains, and strain fluctuations on the electrical performance of graphene. Two-dimensional Raman images of the integrated intensities of the G and D peaks (*I*_G_ and *I*_D_) were used to identify the graphene domain boundaries. The domain boundaries were also identified using Raman images of *I*_D_/*I*_G_ and *I*_2D_/*I*_G_ ratios and 2D spectral widths. Interestingly, the *I*_D_ maps showed that the defects within individual domains significantly increased for the graphene with large domain size. The correlation analysis between the G and 2D peak energies showed that biaxial tensile strain was more developed in the graphene with large domain size than in the graphene with small domain size. Furthermore, spatial variations in the spectral widths of the 2D peaks over the graphene layer showed that strain fluctuations were more pronounced in the graphene with large domain size. It was observed that the mobility (sheet resistance) was decreased (increased) for the graphene with large domain size. The degradation of the electrical transport properties of the graphene with large domain size is mainly due to the defects, tensile strains, and local strain fluctuations within the individual domains.

## Introduction

Large-scale growth of high-quality crystalline graphene is a prerequisite for improving the device performance in graphene-based nanoscale applications. Chemical vapor deposition (CVD) is one of the widely used methods to grow graphene over a large area^1^. In the CVD-grown large-area graphene, however, the formation of graphene domain boundaries, structural defects, and built-in strains cannot be avoided. Consequently, the electrical, mechanical, and thermal properties of polycrystalline graphene are inevitably altered by the presence of the domain boundaries, defects, and strains^2–5^. Defects at the domain boundaries can act as scattering sites of charge carriers, leading to inferior electrical transport properties. For example, the carrier mobility of polycrystalline graphene tends to decrease for a small domain size, suggesting that the charge transport is influenced by scattering at the domain boundaries^6,7^. In contrast, the presence of defects *within* the graphene domain can lead to a reduction in mobility even for a large domain size^8–11^. The tensile strain in the graphene layer induces a decrease in the carrier mobility and an increase in the sheet resistance of the graphene^12–14^. Furthermore, random strain fluctuations and wrinkles also limit the carrier mobility^15,16^. Therefore, the physical properties of polycrystalline graphene are influenced by not only the defects at the domain boundaries but also other defects, strains, and strain fluctuations within the individual domains.

Raman scattering is a powerful tool to probe defects in graphene because the D peak is activated in the presence of disorder^17^. The 2D peak originates from a double-resonance electron-phonon scattering process involved with the electronic dispersion of graphene^17^. Therefore, the 2D peak facilitates the determination of the graphene thickness because the electronic band structure of graphene varies as a function of the number of layers^17^. The symmetric 2D peak of single-layer graphene becomes dispersive with an increase of the number of layers owing to the interlayer coupling, leading to the broadening of spectral width^17,18^. The G peak has *E*_2g_ symmetry involving the C–C bond stretching and originates from a first-order Raman scattering process^17^. Both the G and 2D peaks are sensitive to strain^19,20^. Further, a correlation study of the G and 2D peak energies can explore the residual strain, which would affect the electrical transport properties of graphene^16,21^. Therefore, two-dimensional Raman images of the energies, intensities, and/or widths of the D, 2D, and G peaks scanned over a large area of graphene can provide useful information on the spatial distributions of defects, strains, strain fluctuations, etc. In this study, Raman mapping measurements were carried out on single-layer graphene samples to investigate the influence of defects, strains, and strain fluctuations on the transport properties in the presence of domain boundaries. We observed previously that the sheet resistance (mobility) increased (decreased) for the graphene with a large domain size^11^. Raman images revealed direct evidence that the spatial distributions of defects and strains were different for graphene samples with different domain sizes. Consequently, we observed that the electrical transport properties of the graphene devices could be affected by not only the carrier scattering at the domain boundaries but also the defects, strains, and strain fluctuations within the individual domains.

## Results and Discussion

**Figure 1:**
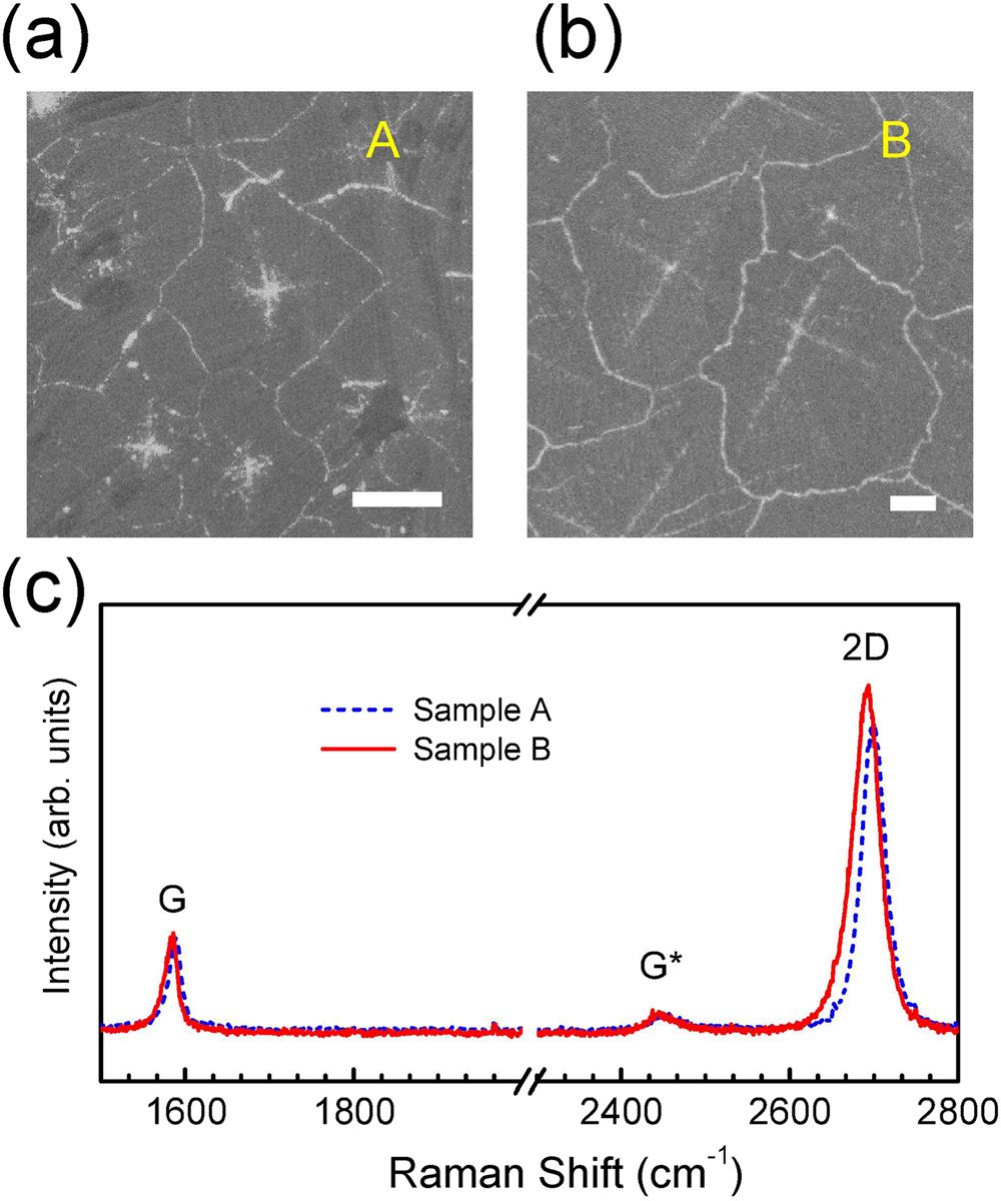
SEM images of samples (**a**) A and (**b**) B. Scale bars are 4 μm. (**c**) Representative Raman spectra of samples A (dashed line) and B (solid line) observed at the excitation wavelength of 488 nm.

Figure 1a and b show the scanning electron microscope (SEM) images of samples A and B, respectively. The samples were mild dry annealed at 200 °C in air for the SEM measurements^11^. Thermal annealing leads to the oxidation of the Cu foil. Since the Cu foil underneath the domain boundaries is more vulnerable to the penetration of O_2_ molecules through defects in graphene, the domain boundaries are distinctly visible as the bright lines in the SEM images^22^. The electrochemical polishing (ECP) treatment combined with an annealing process suppresses the formation of graphene nucleation sites, resulting in an increase in the domain size^23^. Indeed, the average domain size of sample B (∼293 μm^2^) grown using the ECP-treatment followed by the atmospheric pressure H_2_ annealing process is significantly larger than that of the untreated sample A (∼66 μm^2^). Generally, the sheet resistance of polycrystalline graphene is expected to decrease as the graphene domain size increases^2,6,7,24^. In the current study, the sheet resistance was observed to increase for the larger domain size: ∼552 Ω sq^−1^ for sample A and ∼777 Ω sq^−1^ for sample B^11^. Moreover, the mobilities were measured to be approximately 1530 and 910 cm^2^/V·s for samples A and B, respectively^11^. The decrease of the mobility of sample B may be caused by the copper oxide on the surface which was not fully removed due to the suppressed sublimation of copper and oxygen in the atmospheric pressure of H_2_. The copper oxide may induce defects and strain by forming carbon-oxygen bonds and/or small pores on graphene^11^.

Figure 1c shows the representative Raman spectra for samples A and B. The G peak energy for sample A is observed at 1588 cm^−1^. The 2D peak energy for sample A is observed at 2700 cm^−1^, that is consistent with the 2D energy value for an excitation laser energy of 2.54 eV (488 nm)^25^. The 2D peak has a narrow, single Lorentzian line shape and is much more intense than the G peak: a typical signature of monolayer graphene^17^. The weak Raman mode at ∼2450 cm^−1^ corresponds to a multi-phonon response that originates from the combination of the D peak and the longitudinal acoustic phonon^17^. Interestingly, both the G and 2D peaks of sample B are shifted downward in energies compared to the corresponding peak energies of sample A. Moreover, the spectral width of the 2D peak of sample B is much broader than that of sample A. The changes in the G and 2D peak energies and spectral widths in monolayer graphene usually originate from strain and/or strain fluctuations. In order to clarify the differences observed between the samples in more detail, it is necessary to perform spatially resolved Raman measurements over a large area.

Raman intensity changes in CVD-grown monolayer graphene are mainly related to defects, (folded) wrinkles, and overlaps of the graphene layers. Wrinkles are usually formed during the post-growth cooling of graphene on a Cu substrate owing to the difference in thermal expansion coefficients between graphene and Cu^10,26^. Polycrystalline graphene consists of single-crystal domains with different crystallographic orientations. The individual domains are merged together with the formation of defects, wrinkles, and/or overlapped structures at the domain boundaries^10,26,27^. Moreover, defective or atomically smooth connections can be formed at the domain boundaries with flat interfaces^27^.

The D peak is activated for a defective interface but not for an atomically smooth, perfect interface at the domain boundary^27^. The integrated intensities of the G and 2D peaks may not vary regardless of the nature of the interface: either a perfect or a defective flat interface^28^. In contrast, for the multilayer graphene boundary and the folded wrinkle, the integrated intensity of the G peak (*I*_G_) usually increases owing to an increase in the number of graphene layers compared to the surrounding monolayer region^10,29,30^. The integrated intensity of the 2D peak (*I*_2D_) decreases at the wrinkle (not folded) owing to a structural curvature effect, whereas the integrated intensities of the G and D peaks do not change at the wrinkle^26^. At the overlapped bilayer domain boundary, *I*_2D_ depends on the twisted stacking angle between the misoriented monolayer graphene domains^26,31^. Therefore, spatial mappings of the integrated intensity changes in the graphene Raman modes can provide direct spectral evidence on the defects and domain boundary characteristics in polycrystalline monolayer graphene.

**Figure 2:**
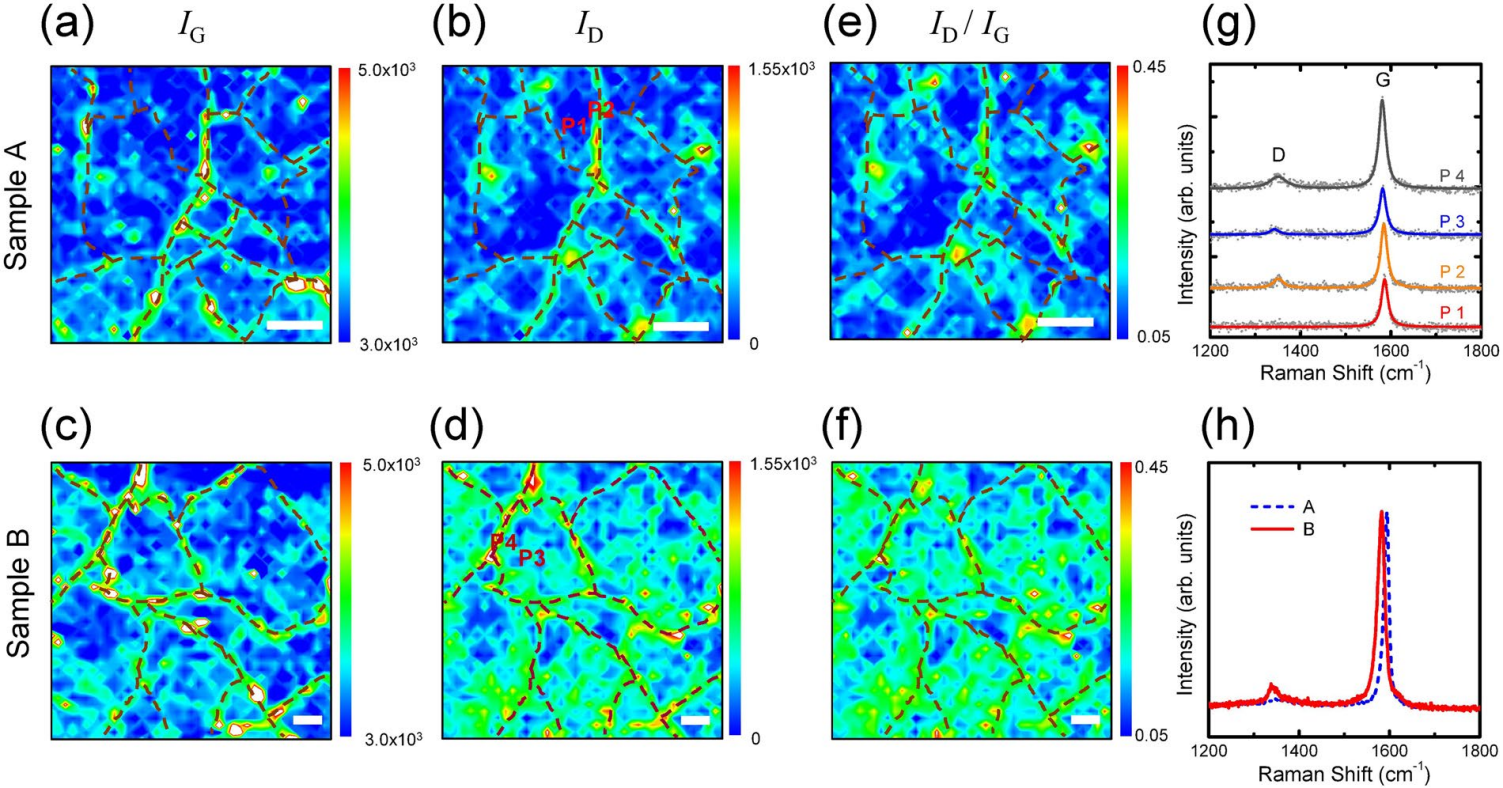
Raman images of *I*_G_ and *I*_D_ for samples (**a**,**b**) A and (**c**,**d**) B. Raman images of *I*_D_/*I*_G_ ratios for samples (**e**) A and (**f**) B. Mapping areas of samples A and B are 20 × 20 and 40 × 40 μm^2^, respectively. Domain boundaries are marked with dashed curves. The Raman images consist of 41×41 pixels with the pixel sizes of 0.5 and 1 μm for samples A and B, respectively. Scale bars are 4 μm. (**g**) Representative Raman spectra within domains (P1 and P3) and at domain boundaries (P2 and P4). Solid circles denote Raman spectral data. Solid lines denote Lorentzian line fits to the data. (**h**) Macro-Raman spectra of samples A and B. The excitation wavelength is 514.5 nm. The spectral coverage for each Raman spectrum is in the range of 1107–2340 cm^−1^.

Figure 2(a–d) show the Raman images of *I*_G_ and *I*_D_ for samples A and B. Note that the samples were *not* mild dry annealed. Interestingly, the *I*_G_ image shows individual domains separated by boundaries with a relatively large *I*_G_ (marked by dashed curves). Furthermore, the *I*_D_ image also exhibits the same boundary structures. The representative Raman spectra in Fig. 2g show that both the G and D peaks are more developed at the boundaries (points P2 and P4 for samples A and B, respectively). Note that both *I*_G_ and *I*_D_ are usually developed at a folded wrinkle or a bilayer domain boundary^10,26^. Therefore, the domain features observed in both *I*_G_ and *I*_D_ images suggest that the enhancements in the G and D peak intensities are mainly attributed to the formation of the graphene domain boundaries. The shape and size of the domains in the Raman mapping images are very similar to those shown in the SEM images (Fig. 1a and b). The enhancement in the integrated intensity ratio of the D to G peaks (*I*_D_/*I*_G_ ratio) is usually related to an increase of defect density, which occurs more at the domain boundaries. It has been reported that the *I*_D_/*I*_G_ ratio does not show a discernable enhancement at the folded wrinkle^10^. Therefore, it is likely that the domain boundary consists of the overlapped graphene. Although sample B shows similar features as sample A in the *I*_D_, *I*_G_ and *I*_D_/*I*_G_ images, two noticeable differences are clearly observed. The average domain size increases significantly from ∼52 μm^2^ (sample A) to ∼254 μm^2^ (sample B) and the D peaks are developed not only at the domain boundaries but also *within* the domains. The macro-Raman spectra in Fig. 2h also confirm that the D peak is significantly increased for sample B. The ECP-treated Cu surface suppresses the formation of nucleation seeds that initiate the growth of large domains. Despite the large domain size, however, the sheet resistance of sample B (∼777 Ω sq^−1^) is higher than that of sample A (∼552 Ω sq^−1^). Therefore, one of the main causes of the increase in the sheet resistance of sample B is attributed to the significant increase of defect density within the domains (Fig. 2d). This result suggests that the transport properties are competitively influenced by not only the size of the domain but also the quality of the domain itself.

The defect density *n*_D_ can be calculated using the following equation^32–34^:

1 $${n}_{{\rm{D}}}\,({{\rm{cm}}}^{-2})=\frac{{10}^{14}}{{\pi }^{2}[{C}_{A}({r}_{{\rm{A}}}^{2}-{r}_{{\rm{S}}}^{2})+{C}_{S}{r}_{{\rm{S}}}^{2}]}\frac{{I}_{{\rm{D}}}}{{I}_{{\rm{G}}}},$$

where *r*_A_ and *r*_S_ represent the radii of the defect-activated and structurally disordered regions, respectively. *C*_A_ depends on the Raman mode given by the ratio of the electron-phonon coupling between two phonons considered and *C*_S_ is a factor that depends on the geometry of the defect for a fixed phonon mode^33,34^. For the D peak, the reported values are approximately given as *C*_A_ = 3.6, *C*_S_ = 2.4, *r*_A_ = 4.1 nm, and *r*_S_ = 2.6 nm^32^. The spatially averaged values of the *I*_D_/*I*_G_ ratios are 0.0703 and 0.1366 for samples A and B, respectively. Thus, the defect densities of samples A and B are estimated to be 1.37 × 10^10^ and 2.64 × 10^10^ cm^−2^, respectively. The defect density of sample B is approximately twice that of sample A.

**Figure 3:**
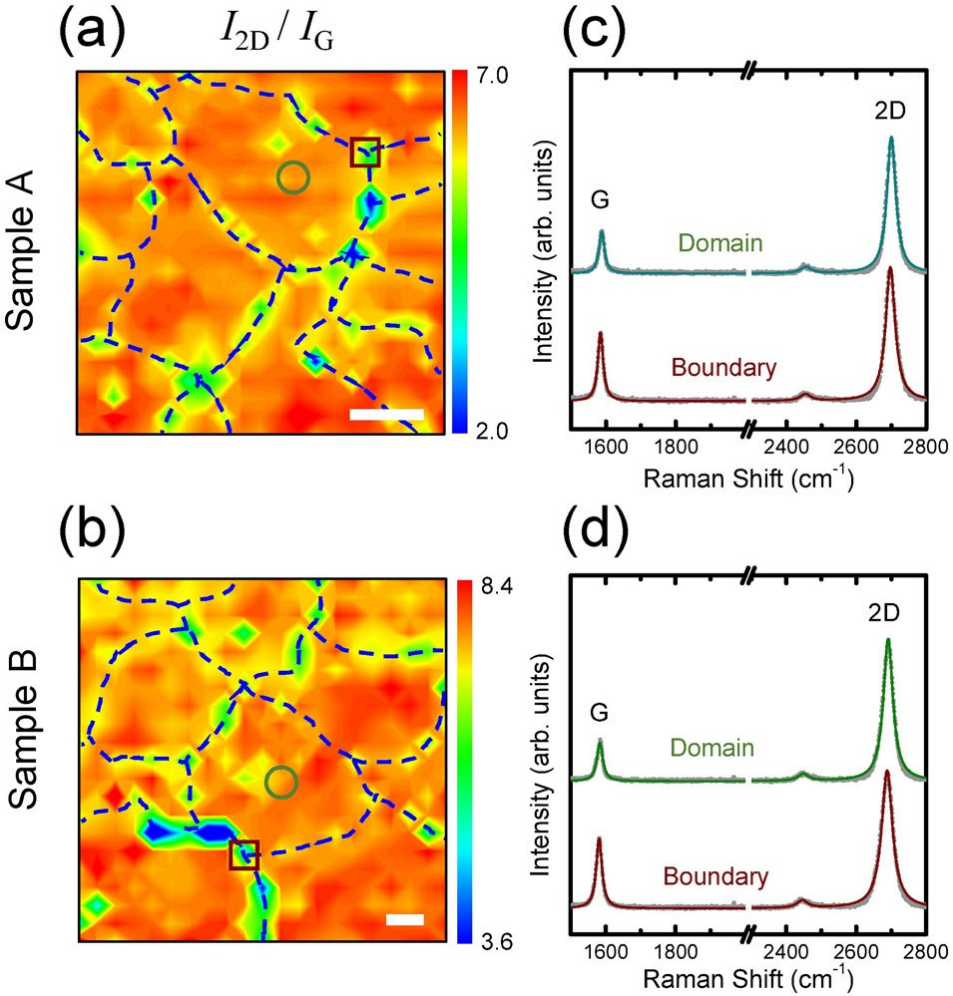
Raman images of *I*_2D_/*I*_G_ ratios for samples (**a**) A and (**b**) B. Mapping areas of samples A and B are 20 × 20 and 40 × 40 μm^2^, respectively. Domain boundaries are marked with dashed curves. The Raman images consist of 21 × 21 pixels with the pixel sizes of 1 and 2 μm for samples A and B, respectively. Scale bars are 4 μm. Representative Raman spectra within domains (open circles in **a** and **b**) and at domain boundaries (open squares in **a** and **b**) for samples (**c**) A and (**d**) B. Solid circles denote Raman spectral data. Solid lines denote Lorentzian line fits to the data. The excitation wavelength is 488 nm. The spectral coverage for each Raman spectrum is in the range of 1475–2807 cm^−1^ wherein the G and 2D peaks are simultaneously observed.

Figure 3a and b show the Raman images of the *I*_2D_/*I*_G_ ratios for samples A and B, respectively. Interestingly, the domain structures are clearly distinguished in the *I*_2D_/*I*_G_ images owing to the significant enhancement in the G peaks at the domain boundaries. As shown in the representative Raman spectra in Fig. 3c and d, the G peak is developed more at the domain boundary than within the domain, whereas *I*_2D_ does not show such a noticeable change. The spatially averaged values of the *I*_2D_/*I*_G_ ratios within domains are approximately 6 and 7 for samples A and B, respectively, indicating that each graphene sample is a single layer^21,31^.

**Figure 4:**
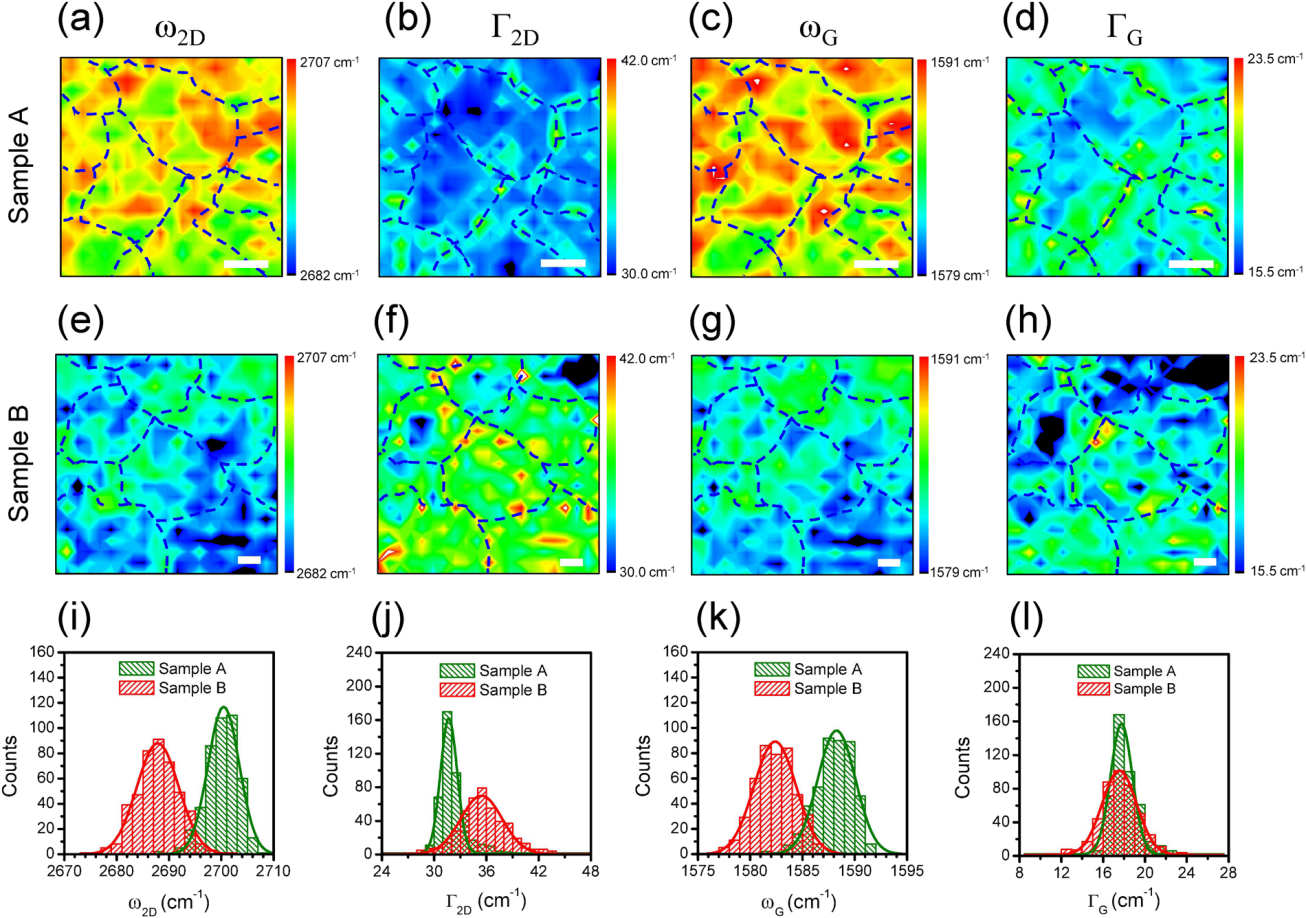
Raman images of *ω*_2D_, Γ_2D_, *ω*_G_, and Γ_G_ for samples (**a–d**) A and (**e–h**) B. Domain boundaries are marked with dashed curves. Histograms of (**i**) *ω*_2D_, (**j**) Γ_2D_, (**k**) *ω*_G_, and (**l**) Γ_G_ for samples A and B. Solid curves in individual histograms are Gaussian fits to the data.

Strain has a significant influence on the transport properties of graphene. For example, the field-effect mobility of graphene tends to decrease in the presence of tensile strain, resulting in the degradation of the device performance^13^. Moreover, random strain fluctuations in graphene can be a dominant source of disorder that limits the scattering of charge carriers, resulting in a decrease in the mobility^15^. The 2D peak energy (*ω*_2D_) and spectral width (Γ_2D_) are mainly sensitive to strain and strain fluctuations, whereas *ω*_G_ and Γ_G_ are sensitive to not only strain but also doping^15,21^. Figure 4a and b show the Raman images of *ω*_2D_ and Γ_2D_ for sample A over the same area shown in Fig. 3a. The Γ_2D_ image of sample A clearly illustrates that the spectral widths are broadened at the domain boundaries, whereas the *ω*_2D_ image does not resolve the domain boundary structure. In comparison, the domain structures are not resolved in both the *ω*_G_ and Γ_G_ images (Fig. 4c and d). The increase in Γ_2D_ at the domain boundary is indicative of the presence of multilayer graphene, whose 2D peak is dispersive^18^. This is consistent with the enhancement of the *I*_D_, *I*_G_, and *I*_D_/*I*_G_ ratio, which indicates the presence of multilayer graphene at the domain boundary (Fig. 2). The multilayer graphene at the domain boundary corresponds most likely to a twisted bilayer structure. In this case, the relative rotation angles between layers are expected to be smaller than 5° because the 2D area intensity is not enhanced at the domain boundary (Fig. 3c and d)^26,31^. For sample B, the Γ_2D_ image does not resolve the domain boundary structure owing to the significant broadening of Γ_2D_ within the individual domains (Fig. 4f). Moreover, similar to the case of sample A, the domain boundaries are not resolved in the *ω*_2D_, *ω*_G_, and Γ_G_ images of sample B (Fig. 4e, g, and h).

In comparison to sample A, the 2D peak energies are significantly shifted downward over the entire area in sample B, indicating the presence of tensile strain. The spatially averaged values of the 2D peak energies for samples A and B are ${\bar{\omega }}_{2{\rm{D}}}$ = 2700.1 ± 3.1 cm^−1^ and 2687.9 ± 4.0 cm^−1^, respectively. Similarly, the downward shifts of the G peaks are also observed for sample B. The average G peak energies for samples A and B are ${\bar{\omega }}_{{\rm{G}}}$ = 1588.0 ± 1.8 cm^−1^ and 1582.4 ± 1.9 cm^−1^, respectively. For sample B, the average values of the 2D and G peak energies are lowered by 12.2 and 5.6 cm^−1^, respectively. Therefore, the ratio of the 2D to G peak shifts is approximately ${\rm{\Delta }}{\bar{\omega }}_{2{\rm{D}}}/{\rm{\Delta }}{\bar{\omega }}_{{\rm{G}}}$ = 2.18. Our result is consistent with the previous report wherein the 2D peak shift under biaxial strain was approximately double the G peak shift^20^. In comparison, the hole doping leads to ${\rm{\Delta }}{\omega }_{2{\rm{D}}}/{\rm{\Delta }}{\omega }_{{\rm{G}}}$ = 0.75 (approximately)^21^. The changes of the G and 2D peak shifts under the biaxial strain ${\varepsilon }_{{\rm{b}}}$ are given by ${\rm{\Delta }}{\omega }_{{\rm{G}}}/{\varepsilon }_{{\rm{b}}}=-62{{\rm{cm}}}^{-1}/{\rm{ \% }}$ and ${\rm{\Delta }}{\omega }_{2{\rm{D}}}/{\varepsilon }_{{\rm{b}}}=-138{{\rm{cm}}}^{-1}/{\rm{ \% }}\,$ ^20^. Under the assumption of biaxial strain, therefore, the average tensile strain in sample B based on the downward shifts of the 2D and G peak energies is estimated to be 0.09%. It is observed that, regardless of biaxial or uniaxial strain, tensile strain leads to an increase (decrease) in the sheet resistance (mobility) of graphene^12–14^. Therefore, the increase (decrease) in the sheet resistance (mobility) of sample B is partly attributed to the tensile strain in sample B as compared to sample A.

Spatially resolved *ω*_2D_ and *ω*_G_ maps provide the variations in the 2D and G peak energies over the scanned areas. Histograms extracted from the distributions of *ω*_2D_ and *ω*_G_ over the scanned areas show that the energy variations are more dispersive in sample B than in sample A (Fig. 4i and k). This is more significant in histograms of Γ_2D_ and Γ_G_ (Fig. 4j and l). Especially, the histogram of Γ_2D_ of sample B is much more dispersive than that of sample A. Furthermore, the spatially averaged width extracted from the spatial distributions of Γ_2D_ is much larger in sample B (${\bar{{\rm{\Gamma }}}}_{2{\rm{D}}}$ = 35.4 ± 3.1 cm^−1^) than in sample A (${\bar{{\rm{\Gamma }}}}_{2{\rm{D}}}$ = 31.7 ± 1.6 cm^−1^). The increase in the spatially averaged value of Γ_2D_ of sample B compared to that of sample A is a signature of a larger magnitude of random strain fluctuations^15^. Therefore, the statistical broadenings of both the 2D peak energy and width distributions as well as the larger average value of the 2D spectral width in sample B suggest that strain fluctuations are more pronounced in sample B than in sample A. These observations provide evidence that the random strain fluctuations are one of the dominant sources of disorder that limits the carrier mobility observed in the electrical transport measurements (approximately 1530 and 910 cm^2^/V·s for samples A and B, respectively).

**Figure 5:**
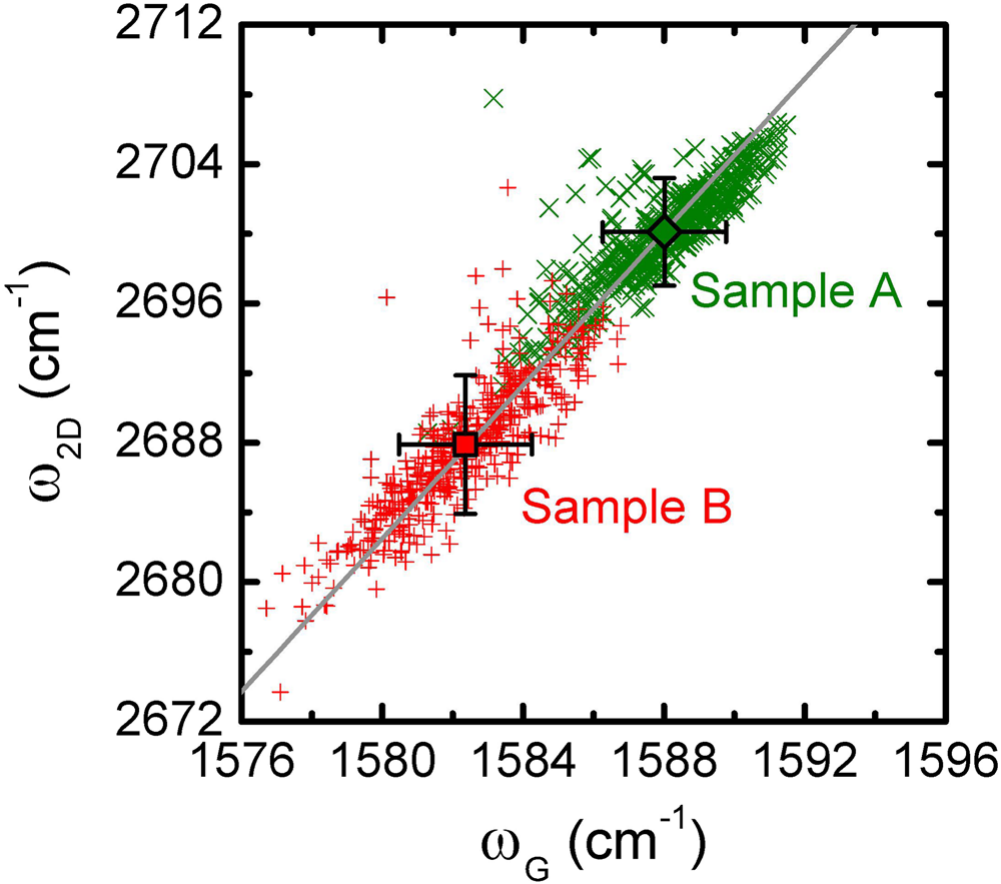
Correlation plots of the G and 2D peak energies (${\omega }_{{\rm{G}}}$,$\,{\omega }_{2{\rm{D}}}$) for samples A (symbols ×) and B (symbols +). The open diamond (square) indicates the spatially averaged value over the area of 20 × 20 μm^2^ (40 × 40 μm^2^) for sample A (B). The slope of the solid gray line is 2.2.

The correlation analysis of *ω*_G_ and *ω*_2D_ provides useful information on strain and can differentiate the effect of strain from that of doping on graphene^20,21,35^. For example, for monolayer graphene under biaxial tension, the strain-induced shift of the 2D peak energy is approximately twice that of the G peak energy^36^. Local strain fluctuations in monolayer graphene can cause spatial variations in the G and 2D peak energies over the graphene layer. Consequently, the correlation points (*ω*_G_, *ω*_2D_) of the G and 2D peak energies scatter along a line with a slope of ${\rm{\Delta }}{\omega }_{2{\rm{D}}}/{\rm{\Delta }}{\omega }_{{\rm{G}}}=$ 2.2 under biaxial strain^16,21^. Similarly, in our case, the correlation points (*ω*_G_, *ω*_2D_) extracted from the Raman images in Fig. 4a and c, e and g for spatial variations in the G and 2D peak energies for samples A and B reveal a linear behavior with a slope of 2.2 (solid gray line in Fig. 5). The slope value of 2.2 suggests that biaxial strain exists in both samples^16,20,21^. The scattered points (*ω*_G_, *ω*_2D_) in sample B are located in the lower part of the correlation plot, clearly indicating that sample B is relatively under tensile strain as compared to sample A.

## Conclusions

Two-dimensional Raman imaging measurements show that the spatial mappings of the spectral variations in the graphene Raman modes provide a unique opportunity to simultaneously probe domain boundary structures, defect distributions, strains, and strain fluctuations, all of which limit the transport properties of polycrystalline graphene. Raman images of not only *I*_D_ and *I*_G_ but also *I*_D_/*I*_G_ and *I*_2D_/*I*_G_ ratios reveal domain boundary characteristics. Interestingly, the Γ_2D_ image also reveals the domain boundary structures. As indicated by the increase of *I*_D_ within the domains, the decrease in ${\bar{\omega }}_{{\rm{G}}}$ and ${\bar{\omega }}_{2{\rm{D}}}$, and the increase in ${\bar{{\rm{\Gamma }}}}_{2{\rm{D}}}$ observed in sample B, defects, tensile strain, and random strain fluctuations are mainly responsible for the increase (decrease) in the sheet resistance (mobility) for graphene with large domain size. Our results provide spectral evidence that domain sizes, defects, and strain-induced disorders are competitively responsible for the scattering of charge carriers that governs the electrical transport properties of graphene devices.

## Methods

### Sample growth and preparation

Single layer graphene films were synthesized on copper (Cu) foils using CVD method. Two different types of Cu foils were used: one was annealed at low pressure without ECP treatment and the other was annealed at atmospheric pressure after ECP treatment. ECP treatment was performed in a mixed solution of phosphoric acid and deionized water. Pre-treated Cu foils were cleansed with deionized water, acetone, and isopropanol in a regular sequence. The Cu foils were heat treated at 1050 °C under hydrogen (H_2_) flow condition for 45 min. The graphene films were synthesized on the Cu foils using methane (CH_4_) and H_2_ gas mixture for 12 min. The sample A (B) was grown on a Cu foil annealed at low (atmospheric) pressure condition of H_2_ without (with) the ECP treatment. After the synthesis of the graphene films, the CVD chamber was rapidly cooled to room temperature.

### Characterization

Spatially resolved Raman scattering measurements were performed on the graphene films transferred onto SiO_2_/Si substrates. The samples were placed on a computer-controlled translational XY stage that was moved in 0.5, 1, or 2 μm intervals along the *x* and *y* directions. Light with an excitation wavelength of either 488 nm or 514.5 nm from an Ar-ion laser was focused on the sample surface through an optical microscope objective lens (100 × /0.9NA) at room temperature. The power of the excitation laser was less than 0.5 mW, at which any laser-induced thermal effects on the samples were not observed. Scattered light from the graphene surface was collected through the same objective lens, dispersed through a spectrometer with a grating of 1200 grooves/mm, and detected using a thermoelectrically cooled charge-coupled device detector. The incident laser polarization was parallel to the scattered light polarization throughout the Raman mapping measurements. The spectral resolution was ∼1 cm^−1^. The spectrometer was calibrated using the known spectral lines of a mercury gas light source.
